# Dermatological

**Published:** 1875-05-15

**Authors:** 


### DERMATOLOGICAL.

*N. Y. Correspondence in American Medical Weekly, May 1, 1875.*

TWO suggestive papers in the department of Dermatology, have recently been presented for the consideration of the societies. The first was read before the Medical Library and Journal Association, by Dr. R. W. Taylor, to the style and scholarship of whose monograph on Syphilitic Lesions of the Osseous System in Infants, the reviewer of the *American Medical Weekly* lately took exception. This subject was the "Ætiology of Infantile Eczema;" and in the outset, he said that he did not wish to state any point dogmatically, but simply to give the results of some of his clinical studies. He alluded to the rheumatic and scrofulous diatheses, but excluded the former from the present consideration, on the ground that rheumatism occurs so rarely in the infant; and when it does, has no appreciable influence on the skin. After some remarks on the characteristics of scrofula, he considered the relation between that affection and eczema. Scrofula engrafts a tendency to inflammation on the skin and mucous membrane, but there is nothing specific about it. Eczema bears the same relation to it as a bronchial or intestinal catarrh for instance. It has, however, an exciting cause also. This is usually only a small furuncle or other insignificant local irritation. The scrofulous form of eczema generally invades extensive surfaces, and is deep-seated, rebellious to treatment, and subject to relapse. Is eczema hereditary—there being no other deviation from normal health in the economy? Hebra answers this question in the negative, and Tilbury Fox in the affirmative. Dr. Taylor agreed with the latter, and showed that icthyosis, xeroderma, nævi, etc., were transmitted in the same way. The explanation of this heredity is, that one portion of the organism alone is stamped with a deviation from the normal condition, and not the whole system. There is, therefore, no proper eczematous diathesis, though a predisposition to cutaneous inflammation is often transmitted from parents to child. He then spoke of the relation between an eczema and other skin diseases in children. The debility following the exanthemata, particularly measles and scarlatina, is especially favorable to eczema, as well as to nasal catarrh, and other evidences of depressed vitality. Any inflammation of the skin predisposes the heart to the same or similar attacks in the future; owing to the impressions made on the blood-vessels, nerves, etc. Vaccination and other local irritations induce a local tendency to inflammation. Sometimes, however, this tendency affects the whole integumentary surface, though the attack is generally most severe at the point of irritation. Finally, the violence and duration of the first local affection have a direct influence on the severity of subsequent attacks. Eczema then, has a local origin in children, but it does not begin spontaneously. It is due primarily to some irritation, and is modified by the scrofulous diathesis, or some of the other conditions alluded to. The frequency of eczema about the head shows this. Among the local exciting causes may be mentioned, the too vigorous use of soap and water, sebaceous accumulations on the head from neglect of cleanliness, pediculi, furuncles, irritating discharges from the eyes, nose or mouth, rough woolen clothing, and the direct action of cold air. Dr. Weisse considered xeroderma, icthyosis and eczema, all manifestations of one tendency, all being found in tissues of very low vitality, and called attention to the influence of dentition in producing eczema. Dr. Taylor explained that he had purposely omitted to speak of such reflex influences as dentition, so as to make his argument as simple as possible.

The second paper to which we have alluded, was on the Relation between the Urine and the Diseases of the Skin, and was read before the Academy of Medicine by Dr. Bulkley, editor of the new *Dermatological Journal*. This subject, he said, was almost totally ignored in the systematic treatise on diseases of the skin, and he had therefore been trying to make some researches in it. Since 1867, he had made 535 analyses of the urine from those suffering from skin affections; but it was only quite recently that he had begun to appreciate the importance of studying the state of the blood, as evidenced by the kidneys, in this class of diseases. He first spoke of the condition of the urine in the exanthemata, particularly erysipelas, scarlatina, measles, and small-pox, and then showed that in urticaria, which seems to have a direct relation to jaundice and rheumatism, there is a deficiency in urates and uric acid. Colchicum restores these, and therefore cures the disease. In erythema, which is also associated with rheumatism, the urine shows the allied condition of lithæmia to be present. Dr. Bulkley has examined the urine of eighty-two persons suffering from eczema, reaching 113 analyses in all, and in not one instance did he find it normal. Two classes of changes were noted: (1) Those denoting acid digestion, the oxalate of lime, etc., being found. (2) Those typical of chronic lithæmia, there being an accumulation of uric or lithic acid, or both.

The specific gravity was usually higher than normal, and the urine was always acid when passed. Albumen was but rarely found. He made twenty-four analyses of the urine of eight persons suffering from psoriasis (which is even more intimately associated with gout), and always found hyper-acidity and oxalate of lime. He also gave the results of his analyses in acne, pruritus and xeroderma, but we have not space for them. In conclusion, he hoped that he had said enough to show that the consideration of the condition of the urine was an important element in the study of dermatology. He thought the German school had done harm by teaching us to disregard the internal condition. Systemic disturbances are almost always present, and it is our duty to correct these, if possible; the examination of the urine affording many valuable hints for therapeutics. The nerve origin of many skin diseases, is entirely compatible with this view of the urinary derangements found in them, and can be readily explained through the medium of the circulation.

